# From integrative disease modeling to predictive, preventive, personalized and participatory (P4) medicine

**DOI:** 10.1186/1878-5085-4-23

**Published:** 2013-11-06

**Authors:** Erfan Younesi, Martin Hofmann-Apitius

**Affiliations:** 1 Department of Bioinformatics, Fraunhofer Institute for Algorithms and Scientific Computing (SCAI), Schloss Birlinghoven, 53754 Sankt Augustin, Germany; 2 Rheinische Friedrich-Wilhelms-Universität Bonn, Bonn-Aachen International Center for IT, Dahlmannstr. 2, 53113 Bonn, Germany

**Keywords:** Disease modeling, Translational bioinformatics, Integrative modeling, P4 medicine

## Abstract

With the significant advancement of high-throughput technologies and diagnostic techniques throughout the past decades, molecular underpinnings of many disorders have been identified. However, translation of patient-specific molecular mechanisms into tailored clinical applications remains a challenging task, which requires integration of multi-dimensional molecular and clinical data into patient-centric models. This task becomes even more challenging when dealing with complex diseases such as neurodegenerative disorders. Integrative disease modeling is an emerging knowledge-based paradigm in translational research that exploits the power of computational methods to collect, store, integrate, model and interpret accumulated disease information across different biological scales from molecules to phenotypes. We argue that integrative disease modeling will be an indispensable part of any P4 medicine research and development in the near future and that it supports the shift from descriptive to causal mechanistic diagnosis and treatment of complex diseases. For each ‘P’ in predictive, preventive, personalized and participatory (P4) medicine, we demonstrate how integrative disease modeling can contribute to addressing the real-world issues in development of new predictive, preventive, personalized and participatory measures. With the increasing recognition that application of integrative systems modeling is the key to all activities in P4 medicine, we envision that translational bioinformatics in general and integrative modeling in particular will continue to open up new avenues of scientific research for current challenges in P4 medicine.

## Review

### Post-genomic era and P4 medicine

**Figure 1:**
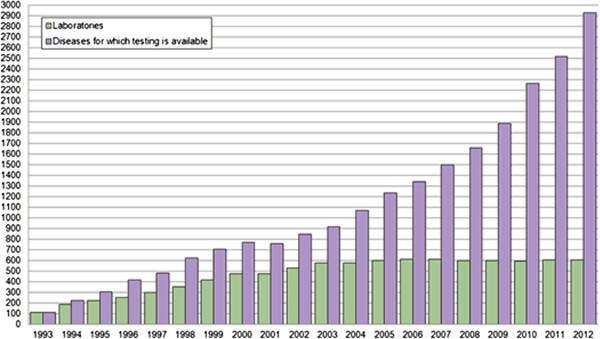
**The rise of genome-based diagnostic applications.** The number of genetic tests for diagnosis of different disease indications - offered by medical laboratories worldwide - has been growing since 1993.

Mendel's studies of inheritance patterns laid the foundation for our current understanding of monogenic or single-gene diseases in human. The laws of Mendelian inheritance, however, could not explain the polygenic or multifactorial inheritance of complex diseases (sporadic vs. familial inheritance). Complex diseases such as cancer or Alzheimer's disease (AD) represent highly heterogeneous clinical states, which reflect combined effects of various genes and their interaction with environmental factors. Analysis and annotation of genomic information has been the main focus of research in the recent past. Since the publication of the Human Genome Project on 26 June 2000, tremendous progress has been made in whole genome sequencing, analysis and interpretation. This progress is reflected in the increasing number of diseases for which genetic testing is available, according to the GeneTests database [1] (Figure 1).

Despite the increasing availability of genetic tests offered by medical laboratories, a fundamental question remains unanswered: how effectively can such molecular data sets be translated into clinical applications, ideally at the point of care, while compatible with patient-centric view of personalized medicine? Although recent efforts such as sequencing human exomes [2], 1000 Genomes Project [3] and Personal Genome Project [4] have been dedicated to characterizing the vast majority of common single-nucleotide polymorphisms (SNPs) and structural variants across the genome, they only provide a one-dimensional view of genome function. Besides, with the advent of new high-throughput technologies such as DNA sequencers, gene expression microarrays and mass spectrometry, other forms of high-dimensional molecular data have emerged that need to be analyzed and used for more accurate diagnosis and treatment prescription.

**Figure 2:**
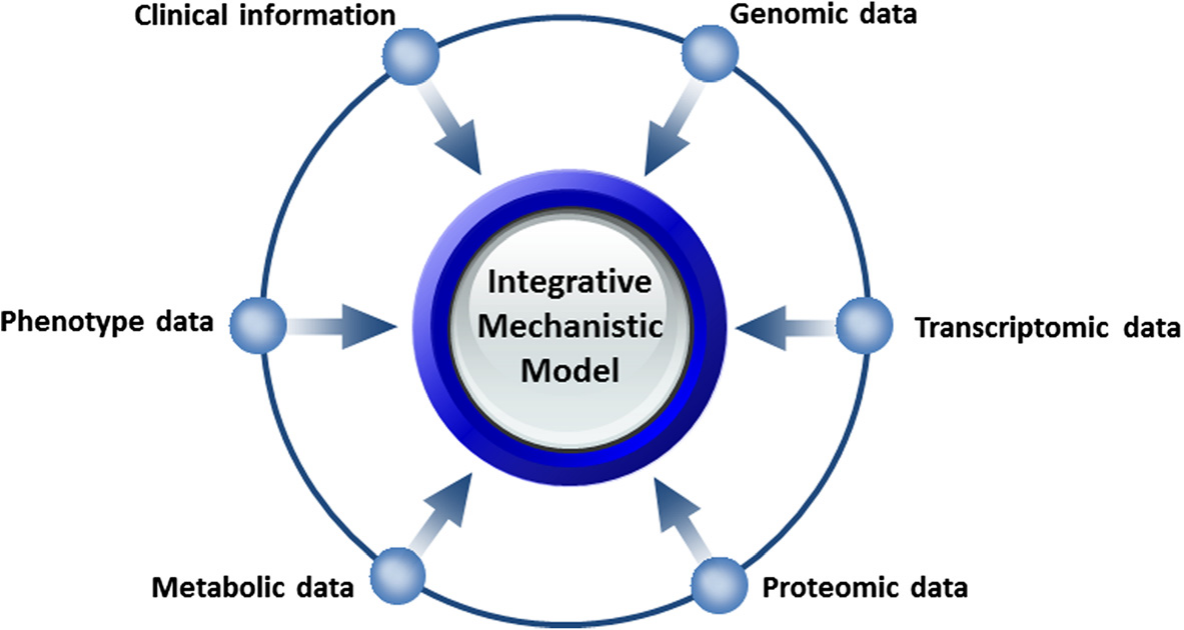
**Disease-specific models at the center of data integration.** Different data types across multiple biological scales can be aggregated and integrated into an integrative mechanistic model.

The realization of P4 medicine promises relies on the ability to manage and integrate different heterogeneous data types across multiple scales from molecular data sets to clinical information and medical history of patients. Handling patients' data and its exchange amongst healthcare information systems has been the focus of informatics endeavors in the realm of personalized medicine [5]. For example, CDISC (Clinical Data Interchange Standards Consortium) brings together leading global biotechnology and pharmaceutical development companies as well as government institutions, academic research centers and other non-profit organizations to develop and support global, platform-independent data standards that enable interoperability of clinical data amongst information systems [6]. Although standardization of data storage and representation is necessary for the future of P4 medicine but it is certainly not enough. The main challenge to be addressed here by translational bioinformatics is the problem of linking *omics* molecular data and affected biological pathways to clinical readouts and epidemiological background of patients with complex diseases. Integration, analysis and interpretation of such heterogeneous data types in the context of complex molecular systems requires an integrative approach to studying systems of biological components, referred to as systems biology. In the post-genomic era, therefore, the greatest challenge is to determine how heterogeneous ‘-omics’ data can be integrated into a coherent holistic model that can explain the disease phenotype in a personalized way. The goal of systems approaches is to decipher the complexity of diseases by integrating all types of heterogeneous biological information into predictive and actionable models (Figure 2). Such models will be central to the premises underlying P4 medicine because they help investigators bridge the ‘translational gap’ between basic and clinical research more efficiently.

Following the recommendations of the EPMA white paper on developing integrative medical approaches [7], we review the current trend in systems modeling and its application to the P4 paradigm. Next, we address the four Ps individually, i.e. predictive, preventive, personalized and participatory, in the context of translational disease modeling and provide scenarios for each case using the latest developments in this area. Finally, we conclude the review with a summary on the emerging implications of integrative disease modeling in P4 medicine.

### Shifting from genome-based to network-based views of human disease

**Figure 3:**
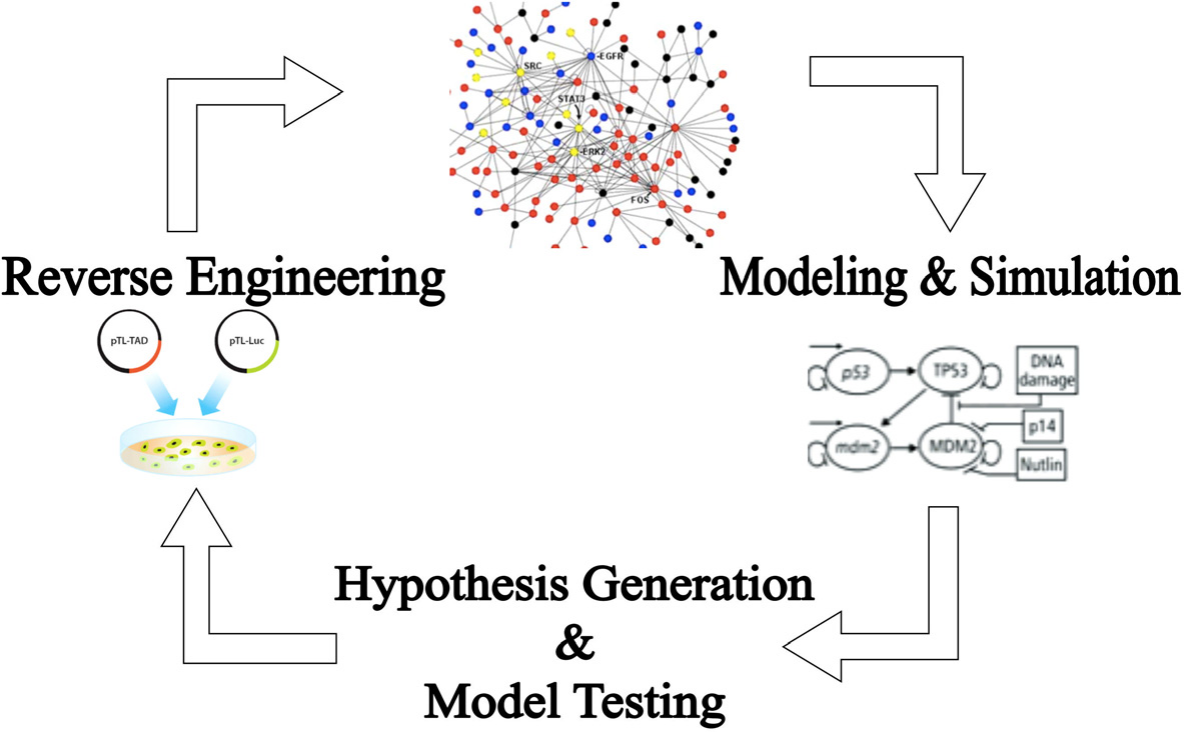
The cycle of systems modeling.

With the paradigm shift in the post-genomic era from single-dimension biological data to multiple dimensions of heterogeneous data, the concept of personalized medicine, which originally defined on the basis of SNPs, is now undergoing a revolutionary change. It is increasingly recognized that analysis of SNPs in isolation does not lead to a complete understanding of complex disease processes and - as a consequence - heterogeneous data must be analyzed and interpreted in an integrated fashion. Systems biology analyses have led to the emergence of new approaches, which take advantage of a network-based integrative view on different biological components involved in pathogenesis of complex diseases rather than a genome-centric single view with no sufficient context. For example, genome-wide association studies (GWAS) have been extensively used to unravel complex associations between genes and diseases at the level of SNPs but they do not provide sufficient context for understanding the complexity of pathological dysregulation involved in the initiation and progression of complex diseases. The knowledge provided by GWAS has a number of limitations that restricts its application, when used slone: the affected genes are difficult to definitively identify and localize, the alteration of gene function by identified SNPs in the context of disease cannot be immediately elucidated and it often remains unknown which particular pathways might be modulated by the SNPs that were found [8-10]. Instead, molecular networks that represent molecular states of the perturbed biological system underlying disease (also known as disease maps) provide a suitable framework for transitioning from ‘descriptive’ to ‘mechanistic’ mode by linking genetic information to disease processes and clinical phenotypes. It is within such a framework that associations amongst biological entities become apparent and a bigger picture of the disease mechanism emerges [11]. To this end, construction of disease-specific molecular maps constitutes the first step towards integrative disease modeling: for example, a computationally tractable interaction map of Parkinson's disease that integrates pathways implicated in Parkinson's pathogenesis has been recently constructed and made openly accessible [12]. However, disease molecular maps only serve as a backbone for the addition of other complementary data from multiple biological scales so that such maps, enriched with genetic variation information, can support researchers in modeling and generating novel hypotheses from those models, which can be further validated in the wet lab (Figure 3).

### Model-driven approach to integrating biomedical knowledge and data

In parallel to the unprecedented growth of experimental data produced by high-throughput technologies, the rate of published knowledge through the biomedical literature has been constantly increasing. Biomedical literature including scientific publications and health records contain valuable information in free-form densely written text. When extracted and converted from unstructured to structured form, literature-derived information can complement data-driven approaches by refining hypotheses generated from high-throughput data sets. Such information helps to distinguish signal from noise when analyzing and interpreting high-throughput data sets [13]. For example, network models of prior biological knowledge have been used to measure the amplitude of perturbation that a given stimulus such as drug treatment or environmental agent induces in a biological system through alterations in gene expression; in this way, the biological impact caused by environmental factors, toxic substances, or drug treatments can be scored and compared [14].

Integration of both experiment-derived and literature-derived information into a single framework has been successfully used for analysis and interpretation of biological mechanisms underlying disease using computational models. Usually modeling biological mechanisms can be driven either by high-throughput experimental data or by prior knowledge of molecular biology published in the literature. The former approach, known as ‘data-driven modeling”, is solely based on the data and no assumption is made about the underlying mechanisms [15]. The limitation of the data-driven approach is, however, the small amount of available quantitative data and the heterogeneity of such data sets. These limitations can be largely compensated with the qualitative statements from the literature [16]. Therefore, the so-called ‘knowledge-driven modeling’ approach makes use of systematically captured expert knowledge, text-mining technologies and semantic resources such as ontologies to build or validate biological networks [17].

**Figure 4:**
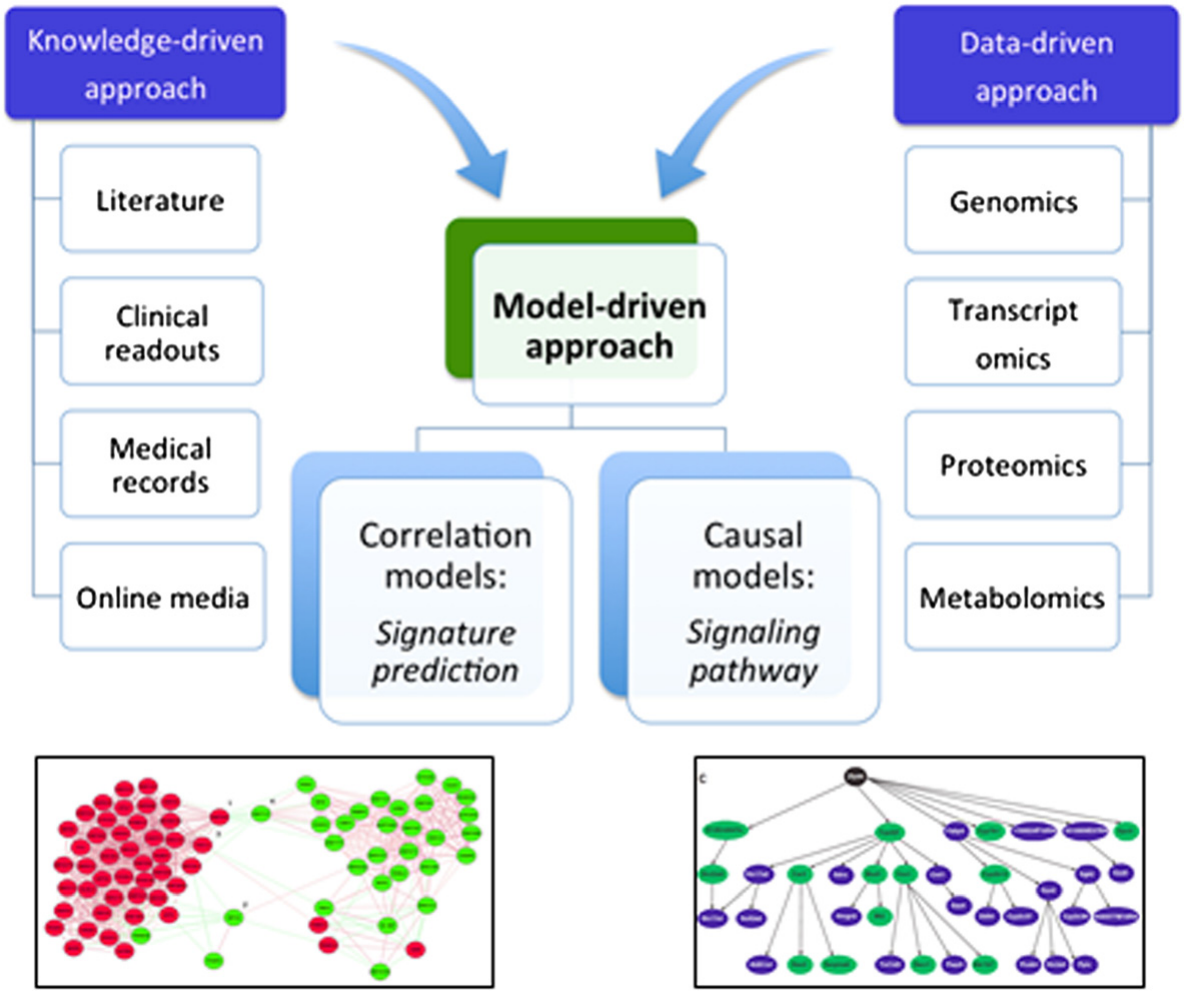
**Model-driven approach to integrating biological data.** Proposed model-driven approach combines biomedical knowledge and data into a single disease model, which could represent correlations (left) or cause and effect (right).

Based on the complementary nature of these two modeling approaches, we propose a hybrid approach - the so-called ‘model-driven approach’ - that combines both data- and knowledge-driven methods. The models generated by this approach could represent correlation or cause and effect, depending on the type of associations between pairs of variables in the network model (Figure 4). Correlation network models are routinely built using high-dimensional data such as protein-protein interactions or gene expression data by establishing pairwise relations (i.e. edges) for all variables (i.e. nodes) but they confound direct and indirect associations (‘is_somehow_related_with’) and do not distinguish between cause and effect. In contrast, causal network models aim at representing response variables and covariates and, thus, the directionality of associations between cause and effect. When relationships between variables represent conditional dependencies (e.g. Given disease symptoms, compute the probabilities of the presence of various diseases), the model is a Bayesian network, which requires information on prior distribution. In the absence of such information, Biological Expression Language (BEL) offers an alternative. BEL is the state-of-the-art causal network modeling language that integrates literature-derived ‘cause and effect’ relationships into a data-driven platform and produces casual network models [18].

We have recently developed BEL-based, computer-processable models representing the physiology of amyloid beta precursor protein (APP); these models represent both the normal and the diseased condition. We have used these models for differential network analysis.

### Central role of integrative modeling in the future of P4 medicine: moving beyond genomics

With the increasingly complex relationship between basic research and clinical application, there is a pressing need to bridge the translational gap from bench to clinic using integrative methods. The mission of ‘translational bioinformatics’ is, therefore, to provide infrastructure and techniques that enable integrative modeling of the whole biological system across multiple scales. Since the objective of P4 medicine is to enable a multitude of purposes in the frame of individualized healthcare including predictive modeling, preventive measures and personalized treatments, a significant amount of personalized data and information should be managed for proper individualized diagnosis and prognosis. As mentioned above, integrative modeling approaches provide a suitable medium for fusion of such data and interpretation of the information derived from such models. We believe that integrative modeling approaches become the major building block of future P4 medicine efforts as their potential in bridging the translational gap is going to be increasingly appreciated and we will soon witness the first examples for model-driven, personalized treatment optimization.

**Figure 5:**
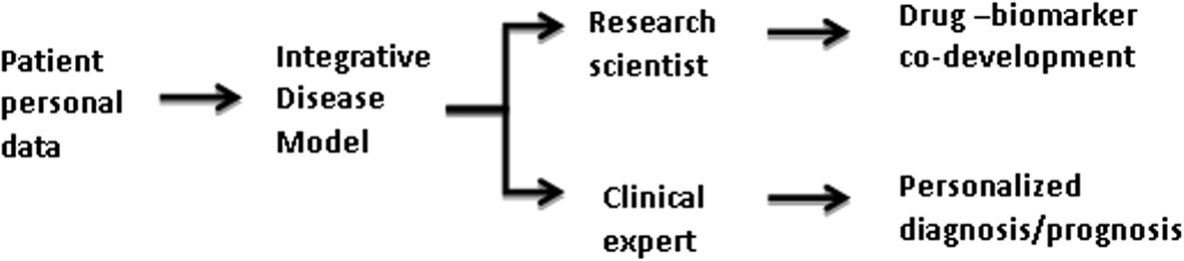
**Decision support options provided by the integrative disease modeling approach.** The integrative modeling approach can provide support to both basic researchers and clinicians in making decisions about diagnostic or prognostic measures.

Application of integrative methods enforces a paradigm shift from the conventional concept of personalized medicine - merely based on genetic makeup of patients (SNPs) - to a modern concept that includes SNPs as one piece of information within a bigger picture amongst other data types. An outcome of this activity is generation of various models that can provide decision support for both researchers and clinicians (Figure 5). For instance, the integration of brain imaging information from each patient into a molecular network model that explains the mode of action of CNS drugs could support clinicians in making decisions about diagnostic or prognostic measures. Similarly, enriched disease models can support the identification of the optimal drugs for targeted treatment of individual patients through the so-called drug-biomarker co-development. Taken together, we envision that general disease models will be personalized and tailored to the need of individual patients through enriching the model with specific patient data.

### Contribution of integrative modeling to P4 medicine

The anticipation that integrative modeling lies at the core of the future P4 medicine raises an important question: how integrative systems models could contribute to solving the real-world problems in predictive, preventive, personalized and participatory areas? In the following, we review the potential contribution of integrative modeling to each P and provide a scenario per P to showcase the applicability of the integrative models to all aspects of P4 medicine.

### Predictive: early diagnosis for the prevention of chronic degenerative diseases

Given the increasingly crucial role of molecular data in the clinical management of patients with complex diseases such as cancer, predictions based on diagnostic biomarkers are emerging as major players in individualized medicine. Biomarkers are defined as indicators of normal biological processes, pathogenic processes, or pharmacological responses to a therapeutic. Diagnostic biomarkers not only support detection of prodromal signs but also determine the progression rout of the disease through indication of stage of the disease or subclinical manifestation of the disease [19].

Predictive diagnostics are needed for early treatment of complex diseases such as Alzheimer's disease or cancer, which present clinical heterogeneity. Conventional diagnostic methods based on measuring just a single parameter encounter the danger of low sensitivity to accurately differentiate patients with highly heterogeneous clinical manifestations. Genome-based molecular fingerprints, including gene expression profiling, have proven successful in linking genome-level events to clinical metrics but they remain far from the clinical application, as they often require invasive sampling and their measurements suffer from lack of consistency. An alternative to such a ‘signature-based approach’ would be a ‘multi-panel approach’ by which gene expression information is linked to proteomics and pathophysiology of disease so that many different types of biomolecules (i.e. a panel of biomarkers) are being associated to pathological processes [20]. Integrative approaches that make use of network models as integration platform have shown a great promise for supporting discovery of highly discerning subsets of molecular biomarkers from the vast combinatorial space of molecular candidates [21]. For instance, the integration of gene expression profiles obtained from primary breast tumors in a protein interaction network model led to identification of sub-network biomarkers that represent metastatic tumor progression [22].

**Figure 6:**
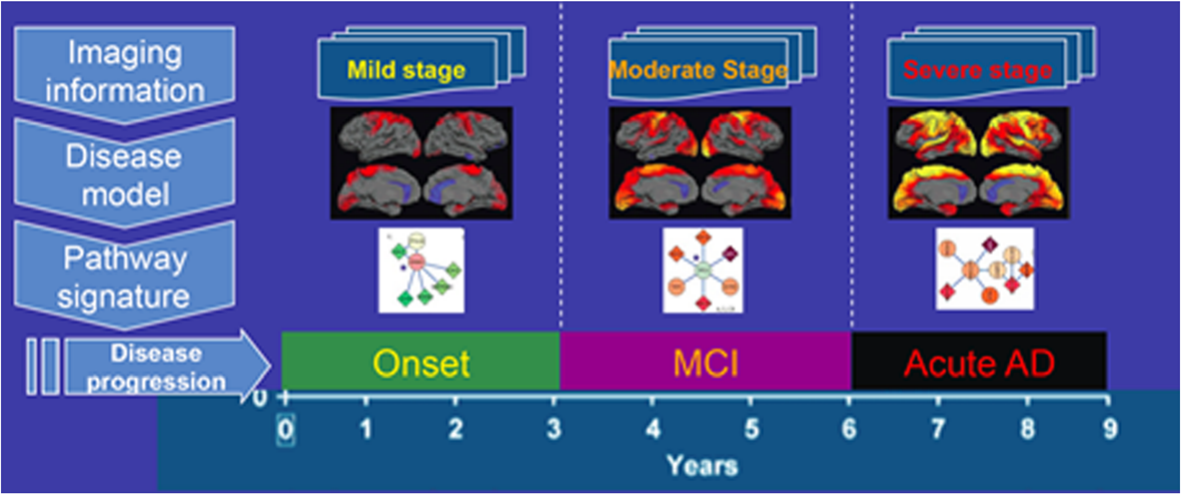
**Modeling staging mechanism in Alzheimer's disease.** Schematic representation of the proposed workflow for translation of imaging information into stage-specific disease maps.

Although predictive diagnostics are conventionally represented by genome-based methods and tests, imaging markers are emerging as an integral part of medical diagnosis, particularly for diagnosis of neurological disorders. Hence, translating imaging readouts to diagnostic molecular biomarkers is expected to be extremely valuable. To this end, we have developed an algorithm that incorporates the diagnostic knowledge of imaging and potential protein biomarkers specific to Alzheimer's disease into a brain-specific protein interaction network [23]. As a result, three network models representing disease stages were generated and subjected to pathway analysis (Figure 6). The results indicated that, in contrast to the widely accepted amyloid pathway hypothesis, particular pathways are causally related to the disease mechanism. This was not appreciated before.

### Preventive: reducing the likelihood of disease and disability

The primary goal of preventive measures against complex diseases is to identify at-risk individuals long before the development of disease symptoms (for instance, individuals at risk of developing Alzheimer's disease) so that preventive treatments can be planned. Accordingly, preventive biomarkers aim at screening a population and stratifying individuals at a high risk of developing disease by measuring the association between their molecular profile (e.g. gene expression or genetic variation) and disease phenotype. However, finding efficient preventive biomarkers is a non-trivial task that requires characterization of composition, distribution and function of key molecules involved in the disease mechanism.

**Figure 7:**
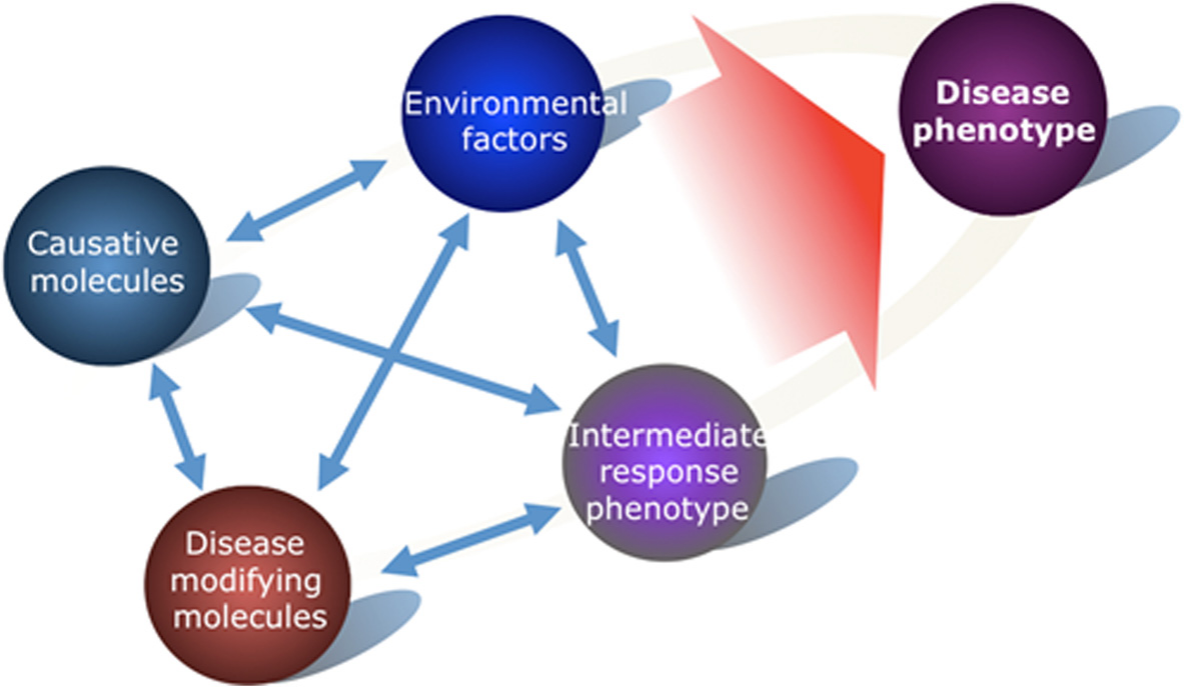
**Deterministic factors involved in shaping the disease phenotype.** The disease phenotype is the result of interactions between causative molecules, environmental factors and disease-modifying molecules.

Computational modeling approaches have been used already to assist the process of stratification biomarker discovery for cardiovascular diseases [24]. Recently, an interesting area for application of computational models to disease prevention is emerging that uses mechanistic models to reveal interdependencies between the disease and risk mechanisms (Figure 7). Such interdependencies are reflected in comorbidities that co-occur with the disease and increase the disease risk.

In the following, we present a scenario that shows therapeutic prevention of diabetes can reduce the risk of developing Alzheimer's disease in at-risk populations.

Epidemiological findings indicate that type II diabetes mellitus is linked to developing and exacerbating AD pathology [25,26] so that Alzheimer's has been even proposed by some authors to be ‘type III diabetes’ [27,28]. Very recently, a 9-year prospective study on 3,069 elderly adults without dementia demonstrated that patients who suffered from diabetes had significantly worse cognitive decline in comparison with those who did not have the disease, suggesting the contribution of diabetes mellitus severity to accelerated cognitive impairment [29]. On the other hand, pharmacological studies provide evidence that application of anti-diabetes drugs confer protective effects to demented brains. In mouse models of Alzheimer's disease, application of an anti-diabetic drug called extendin-4 reversed insulin pathology and improved cognition significantly [30]. In another study, it was shown that the diabetes drug Liraglutide prevents key neurodegenerative developments in a mouse model of Alzheimer's disease [31]. Rosaglitazone, another anti-diabetic agent, also showed beneficial effects for dementia treatment when administered at low dose [32]. In spite of such growing evidence, the molecular mechanism underlying this protective effect is still unclear and thus, aggregation and analysis of disparate knowledge on this topic in the frame of a unified computational model may facilitate the mission of finding a preventive treatment for Alzheimer's disease. In this direction, we have recently proposed a mechanistic model for genomic hormone interactions underlying dementia, which not only reveals the possible molecular connections between the insulin signaling pathway and learning/memory functions but also explains the preventive mode of action by serendipitous off-target effects of several approved drugs [33].

### Personalized: targeted therapy as an emergent trend in drug discovery and development

**Figure 8:**
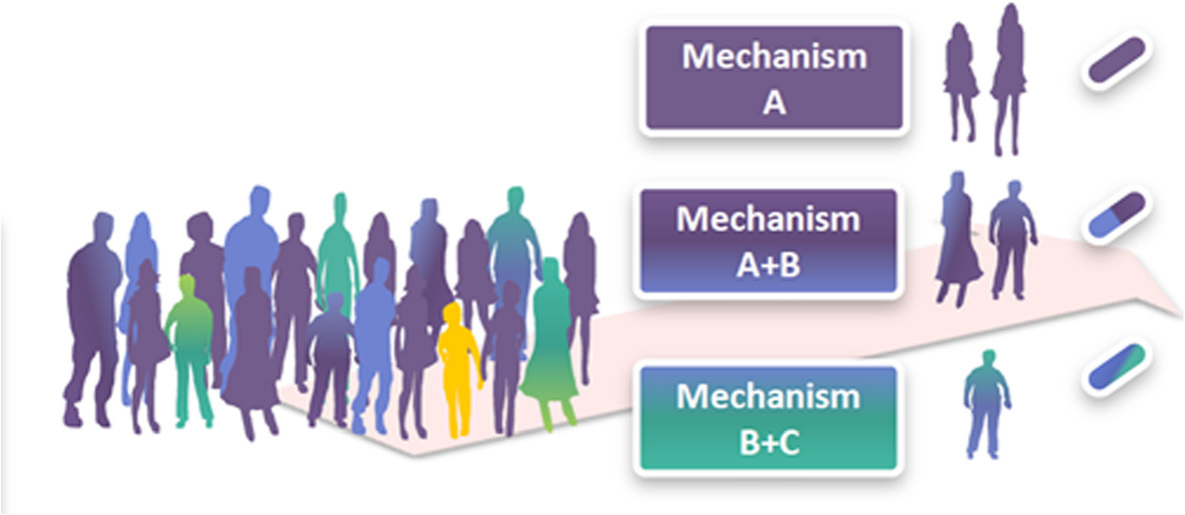
Stratification of patient population based on disease mechanism.

The concept of personalized medicine was first coined in the context of genetics but its definition has been extended beyond genomics to the customization of healthcare measures to individual patients [34]. The ultimate goal of personalized medicine is to use integrative data to better target the delivery of healthcare and treatments to individuals [35]. Therefore, strategies and methods are required to enable classification of individuals into subpopulations based on their susceptibility to disease or response to treatment (Figure 8). Accordingly, successful stratification of individuals is the key to the following preventive and therapeutic interventions [36].

Prognostic and predictive biomarkers are considered to be the key contributors to the success of personalized medicine. Prognostic biomarkers stratify patients undergoing a specific therapy based on the risk of disease progression whereas predictive (stratification or companion) biomarkers identify patients who are more likely to successfully respond to specific therapeutic intervention. A major drawback in delivering right pharmacological treatment to right patients is that the current therapeutic options are based on the ‘one-size-fits-all’ paradigm and thus not appropriate for stratification of responders from non-responders to treatment. Due to this fact and also because of a couple of other reasons, including the pressing need for better safety and efficacy indices and the need to control increasing healthcare expenditures, a new trend is emerging in pharmaceutical businesses that shifts from mass therapies towards targeted therapies based on so-called companion biomarkers [37]. Despite previous success stories of companion biomarker applications, a significant gap exists between the R&D expenditure and available clinically validated biomarkers. Therefore, better understanding of the mode of action of drugs as well as the alternative pathways that can reduce therapeutic effects will be crucial to discovery of novel companion biomarkers.

We have recently reviewed current biomarker-discovery technologies highlighting challenges and opportunities for oncology biomarker discovery with the emphasis on computational integrative modeling approaches as an emerging trend in biomarker prediction [38]. The overall trend indicates that there is a move away from correlative biomarkers towards causative biomarkers. Thus, the aim of next-generation integrative models is to capture causal relationships between candidate biomarker and clinical outcome. A prime example is development of predictive biomarker content for identification of patients with ulcerative colitis who could be potential responders to targeted anti-TNF therapy with Infliximab [39]. Based on the prior knowledge in the literature, a causal network model was constructed that describes mechanistic knowledge underlying ulcerative colitis in the form of the ‘cause-relationship-effect’ pattern. Next, gene expression profiles of responders and non-responders were incorporated into the causal model and a mechanistic strength value was calculated on the gene expression network activity signature of TNF signaling for each patient in the population. The model demonstrated that non-responders have different TNF signature compared to responders, which was due to sustained TNF-like downstream signaling in non-responders after treatment with Infliximab, controlled by alternative upstream controllers.

### Participatory: enriching models with patient-centric information and experiences

In parallel to the concepts of personalized medicine and targeted therapy, patient-centric medicine is developing based on patient-oriented research. The aim of patient-oriented research is to identify the best treatment strategy guided by disease heterogeneity diagnostics [40]. The internet has facilitated participation of individual patients in the healthcare through sharing their experiences in blogs and other social media. Indeed, online patient communities represent a true model for participatory medicine. For instance, PatientsLikeMe [41] is an online patient community that provides robust methods for patients to share their experiences on effectiveness of treatments. Since such virtual environments act as repositories of self-reported data on the nature of disease, treatment response and side effects, they have the potential to be used for refining the existing drug-response models or optimizing clinical outcomes. For instance, Wicks et al. (2011) used the self-reported data of 348 ALS patients - posted on the PatientsLikeMe website - and based on these reports demonstrated that treatment with lithium carbonate has not been beneficial for the patients [42].

Together with blogs and online resources, electronic health records (EHRs) present valuable sources for integrating patient data into disease models. EHRs have been used to stratify neuropsychiatric patient cohorts based on complete phenotypic profiles than the conventional primary diagnosis [43]. Very recently, we have applied our Alzheimer's disease ontology (ADO) to 650 AD patients' health records and demonstrated that four major comorbidities reported in these records also link mechanistically to the pathology of AD [44]. There are emerging initiatives like the ‘Quantified Self’ initiative [45], which will lay the foundation for the concept of virtual patient. The virtual patient is a model that can be compared (and populated/parameterized) by individuals. Quantified Self initiative collects self-tracking tools and applications in one place and facilitates finding and access to self-recording/self-reporting application softwares.

In summary, the integration of medical data from participating patients into disease models is imperative if these models are to be used for patient stratification.

### Emerging applications of integrative disease modeling in P4 medicine

With the increasing recognition that integrative systems modeling will play a major part in all future activities in P4 medicine, we envision that translational bioinformatics in general and systems modeling in particular offer more solutions to current challenges in P4 medicine. In the following, we provide an outlook on the emerging trend and future prospects in this area.

#### Molecular-based classification of diseases and their subtypes

The current classification of diseases is based on anatomical, symptomatic and epidemiological criteria, which does not take the etiological mechanism into account. This has led to the problem of misdiagnosis due to the overlap of symptoms. Consider Parkinson's disease with dementia and Alzheimer's disease dementia. Since classification of these two neurodegenerative diseases is merely based on their clinical signs, early diagnosis of Parkinson's disease (PD) is hampered. The extent of clinical overlap between these two conditions is so much greater than chance that even some authors propose that AD and PD belong to a spectrum of neurodegenerative disorders with common disease mechanism but triggered by different etiological factors [46]. However, at the molecular level, these two conditions can be distinguished based on diagnosis between synucleinopathy from tauopathy. Thus, integrative disease modeling provides a framework for incorporation of both conventional reductionism and non-reductionism approaches and allows for re-definition of the current nosology [47].

**Figure 9:**
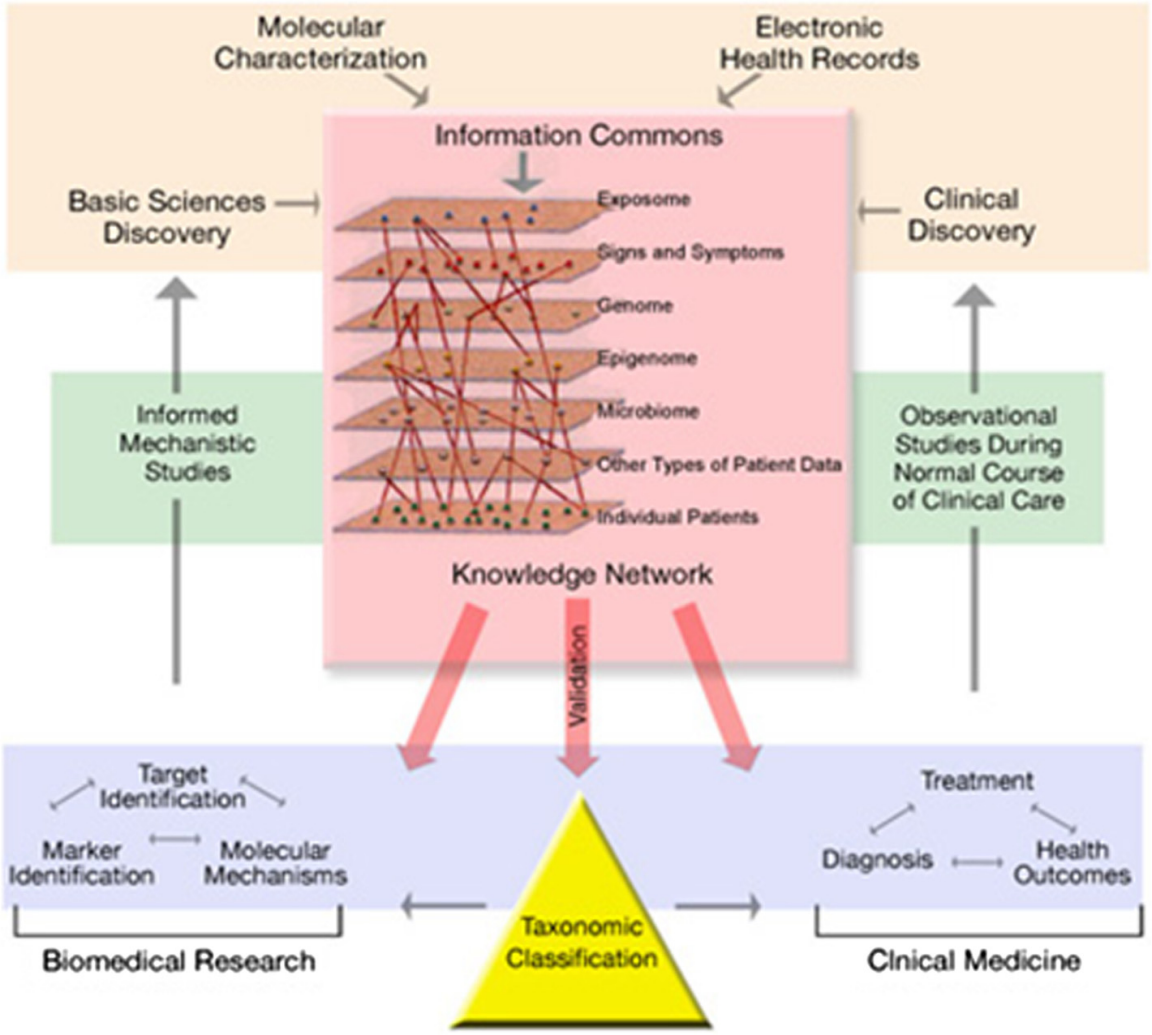
Architecture of the knowledge network information system proposed by NAS committee.

In 2011, National Academy of Sciences committee recommended NIH to develop a new taxonomy of diseases based on their underlying molecular and environmental causes rather than on physical signs and symptoms [48]. The idea is to create a so-called Knowledge Network information system that integrates molecular data, medical histories and health outcomes of individual patients (Figure 9).

In 2012, the Innovative Medicines Initiative (IMI) - Europe's largest public-private partnership aiming to improve the drug development process - launched a call that addresses the topic of developing an aetiology-based taxonomy for human diseases. The proposed methodology for this topic that was submitted by Fraunhofer SCAI and named AETIONOMY, was selected by IMI consortium in which we propose a knowledge-based approach to modeling neurodegenerative disease mechanisms underlying Alzheimer's and Parkinson's disease and signature-based classification of syndromes belonging to these disorders. The aim of AETIONOMY is to come up with a new mechanism-based classification system for AD and PD so that mixed pathologies could be unequivocally distinguished, new features or classes could be easily accommodated in the taxonomy, and using this information for drug and biomarker discovery could be facilitated.

#### Predictive drug re-purposing and modeling polypharmacology effect

Low productivity of drug discovery pipelines in recent years has been largely attributed to insufficient efficacy of failed drugs. Failure of drugs in phases II and III clinical trials in fact reflects the poor understanding of the mode of action of such drugs at the molecular level. It is becoming increasingly evident that approved drugs have more than one target and this ‘multi-targeting’ nature of drugs could have both positive and negative consequences in terms of increased efficiency of therapeutic effect or adverse reactions, respectively. Since approved drugs have already passed safety tests in clinical trials, they can be re-used for new therapeutic indications; however, efficacy still has to be shown for the repositioned drug. At this point, the re-purposing strategy can be linked to efficacious targeted therapy for personalized medicine applications [49]. The recent approval of crizotinib for treatment of non-small cell lung cancer (NSCLC) that was repositioned from anaplastic large-cell lymphoma therapy is a good example of linking drug re-purposing to personalized medicine. The repositioned drug is accompanied by a diagnostic test for stratification of NSCLC patient subsets [50].

Usually, novel disease indications for known drugs have been discovered by serendipity. It is only recently that computational and systems approaches have been employed for inferring and predicting drug repurposing. These approaches can be grouped into target-based (similar binding sites) or disease-based (similar mechanisms) [51]. The rational design of ligands acting on multiple targets requires that such ligands bear certain molecular properties. We have recently devised a machine learning method that successfully classified approved drugs to NDD and non-NDD drugs based on a set of ten pharmacological properties [52]. Integration of drug pharmacological data and target structural features into tissue- or organ-specific disease models will support the systematic identification of new opportunities (‘re-purposing’) for existing drugs.

#### Integrative modeling in support of drug development decision making

The goal of integrative disease modeling is to provide a framework for hypothesis generation. Thus, it is expected that modeling predictions provide many inputs into the decision-making process in the pharmaceutical industry [53]. Since the main reasons for drug failures in the clinic are efficacy and safety, target identification and validation are the most important steps in drug development. Disease-specific computational models, when enhanced and enriched with efficacy data, can assist scientists to construct a more robust rationale in support of the main hypothesis compared to competing hypotheses. Based on this approach, a likelihood function can be developed and applied to proof-of-concept decision making so that the late-phase drug failures caused by lack of efficacy could be prevented [54]. Such an integrative model will summarize the evidence from multiple data sources that supports competing hypotheses on the basis of observed data at any time point.

#### Comparative modeling of rodent and human pathobiological mechanisms

High rate of drug failures in general and recent failures of Alzheimer's therapies in phase III of clinical trials in particular, despite substantial support from animal studies, raise the concern whether animal models of chronic complex diseases reliably inform human conditions. Several factors including insufficient statistical power, inadequate internal validity of animal experiments (methodological flaws) and lack of external validity (differences between animals and humans in the pathophysiology of the disease) have been reported to be responsible for the translational failure of apparently promising animal studies [55].

Reduced external validity of animal studies, including insufficient similarity to the human condition and ignorance of comorbidities, can partially explain the mechanistic issues behind the poor efficacy of failed drugs. For example, a systematic study evaluating the mimicry precision of human inflammation by mouse models showed that inflammatory responses in murine models poorly correlated with the human conditions [56].

In the case of chronic, progressive diseases such as neurodegenerative disorders, current research has mainly relied on injury-induced models that do not recapitulate the progressive, degenerative nature of the disease in humans. Reasons for this could be attributed to fundamental differences in neural circuitry between human and rodents as well as different molecular mechanisms in man from animal models [57]. The fact that mouse models of human neurodegenerative diseases have largely failed to predict the efficacy of clinical trials calls for more rigorous validation of predictions made with the help of mouse models using alternative strategies [58]. For instance, in response to the Species Translation Challenge by sbv IMPROVER [59], we sought to systematically model and mechanistically compare neuroinflammatory pathways between human and mouse in the context of Alzheimer's disease. Differential pathway analysis on both models revealed unique and segregating pathways in human and mouse.

Integrative disease modeling could offer a powerful approach to increasing the probability of predicting efficacy of animal data by providing comparative mouse-human computational models that compute the extent to which molecular details of animal models match with the corresponding molecular mechanisms in human subjects. Ideally, such a comparative study should take the multiple biological scales from cell and tissue types to genes, proteins and pathways into account so that it clearly reveals anatomic and mechanistic differences.

## Conclusions

Integrative *in silico* disease modeling is emerging as a new paradigm at the core of translational research, which prepares the ground for transitioning from descriptive to mechanistic representation of disease processes. Given the tremendous potential of integrative disease modeling in supporting translation of biomarker and drug research into clinically applicable diagnostic, preventive, prognostic and therapeutic strategies, it is anticipated that computer-readable disease models will be an indispensable part of future efforts in the P4 medicine research area.

## Competing interests

The authors declare that they have no competing interests.

## Authors’ contributions

EY conceived of the study, designed the methodology, performed analyses and drafted the manuscript. MHA revised the manuscript critically. Both authors read and approved the final manuscript.

## References

[B1] GeneTests Databasehttp://www.genetests.org

[B2] Tennessen JA Bigham AW O'Connor TD Fu W Kenny EE Gravel S McGee S Do R Liu X Jun G Kang HM Jordan D Leal SM Gabriel S Rieder MJ Abecasis G Altshuler D Nickerson DA Boerwinkle E Sunyaev S Bustamante CD Bamshad MJ Akey JM Evolution and functional impact of rare coding variation from deep sequencing of human exomes Science 2012 337 64 69 10.1126/science.1219240 22604720 PMC3708544

[B3] A Deep Catalog of Human Genetic Variationhttp://www.1000genomes.org

[B4] Personal Genome Projecthttp://www.personalgenomes.org

[B5] Shabo A Scarpa M Mandel S Bridging the informatics gap between bench and bedside: implications to neurodegenerative diseases Neurodegenerative Diseases: Integrative PPPM Approach as the Medicine of the Future 2013 Berlin: Springer 301 308

[B6] Clinical Data Interchange Standards Consortiumhttp://www.cdisc.org/

[B7] Golubnitschaja O Costigliola V EPMA: **General report & recommendations in predictive, preventive and personalised medicine 2012: white paper of the European association of predictive, preventive and personalised medicine.** EPMA J 2012 3 14 10.1186/1878-5085-3-14 23116135 PMC3485619

[B8] Chen Y Zhu J Lum PY Yang X Pinto S MacNeil DJ Zhang C Lamb J Edwards S Sieberts SK Leonardson A Castellini LW Wang S Champy MF Zhang B Emilsson V Doss S Ghazalpour A Horvath S Drake TA Lusis AJ Schadt EE Variations in DNA elucidate molecular networks that cause disease Nature 2008 452 429 435 10.1038/nature06757 18344982 PMC2841398

[B9] Emilsson V Thorleifsson G Zhang B Leonardson AS Zink F Zhu J Carlson S Helgason A Walters GB Gunnarsdottir S Mouy M Steinthorsdottir V Eiriksdottir GH Bjornsdottir G Reynisdottir I Gudbjartsson D Helgadottir A Jonasdottir A Jonasdottir A Styrkarsdottir U Gretarsdottir S Magnusson KP Stefansson H Fossdal R Kristjansson K Gislason HG Stefansson T Leifsson BG Thorsteinsdottir U Lamb JR Genetics of gene expression and its effect on disease Nature 2008 452 423 428 10.1038/nature06758 18344981

[B10] Witte JS Genome-wide association studies and beyond Annu Rev Publ Health 2010 31 9 20 10.1146/annurev.publhealth.012809.103723 PMC3997166 20235850

[B11] Tegner J Compte A Auffray C An G Cedersund G Clermont G Gutkin B Oltvai Z Stephan K Thomas R Villoslada P Computational disease modeling - fact or fiction? BMC Syst Biol 2009 3 56 10.1186/1752-0509-3-56 19497118 PMC2697138

[B12] Fujita KA Ostaszewski M Matsuoka Y Ghosh S Glaab E Trefois C Crespo I Perumal TM Jurkowski W Antony PM Diederich N Buttini M Kodama A Satagopam VP Eifes S Del Sol A Schneider R Kitano H Balling R Integrating pathways of Parkinson's disease in a molecular interaction map Mol Neurobiol 2013 1 15 10.1007/s12035-013-8489-4 PMC4153395 23832570

[B13] Roberts PM Mining literature for systems biology Brief Bioinform 2006 7 399 406 10.1093/bib/bbl037 17032698

[B14] Martin F Thomson TM Sewer A Drubin DA Mathis C Weisensee D Pratt D Hoeng J Peitsch MC Assessment of network perturbation amplitudes by applying high-throughput data to causal biological networks BMC Syst Biol 2012 6 54 10.1186/1752-0509-6-54 22651900 PMC3433335

[B15] Janes KA Yaffe MB Data-driven modeling of signal-transduction networks Nat Rev Mol Cell Biol 2006 7 820 828 10.1038/nrm2041 17057752

[B16] Chang R Shoemaker R Wang W A novel knowledge-driven systems biology approach for phenotype prediction upon genetic intervention IEEE/ACM Trans Comp Biol Bioinform 2010 8 1170 1182 10.1109/TCBB.2011.18 PMC3211072 21282866

[B17] Ananiadou S Kell DB Tsujii JI Text mining and its potential applications in systems biology Trends Biotechnol 2006 24 571 579 10.1016/j.tibtech.2006.10.002 17045684

[B18] The Biological Expression Languagehttp://www.openbel.org

[B19] Mayeux R Biomarkers: potential uses and limitations NeuroRx 2004 1 182 188 10.1602/neurorx.1.2.182 15717018 PMC534923

[B20] Wang K Lee I Carlson G Hood L Galas D Systems biology and the discovery of diagnostic biomarkers Dis Markers 2010 28 199 207 10.1155/2010/130861 20534905 PMC3021550

[B21] Dudley JT Butte AJ Identification of discriminating biomarkers for human disease using integrative network biology Pac Symp Biocomput 2009 27 38 19209693 PMC2749008

[B22] Chuang HY Lee E Liu YT Lee D Ideker T Network-based classification of breast cancer metastasis Mol Syst Biol 2007 3 140 17940530 10.1038/msb4100180 PMC2063581

[B23] Younesi E Hofmann-Apitius M Bridging clinical knowledge to molecular events through integration of imaging biomarkers and protein interactions in Alzheimer’s disease http://www.iscb.org/archive/conferences/iscb/cms_addon/conferences/ismbeccb2011/posterlist8ba3.html?cat=X

[B24] Syed Z Stultz CM Scirica BM Guttag JV Computationally generated cardiac biomarkers for risk stratification after acute coronary syndrome Sci Transl Med 2011 3 102ra95 21957173 10.1126/scitranslmed.3002557

[B25] Sims-Robinson C Kim B Rosko A Feldman EL How does diabetes accelerate Alzheimer disease pathology? Nat Rev Neurol 2010 6 551 559 10.1038/nrneurol.2010.130 20842183 PMC3199576

[B26] Ott A Stolk RP van Harskamp F Grobbee DE Breteler MM Diabetes mellitus and the risk of dementia: The Rotterdam Study Neurology 1999 53 1937 1942 10.1212/WNL.53.9.1937 10599761

[B27] de la Monte SM Wands JR Alzheimer's disease is type 3 diabetes-evidence reviewed J Diabetes Sci Technol 2008 2 1101 1113 19885299 10.1177/193229680800200619 PMC2769828

[B28] Akter K Lanza EA Martin SA Myronyuk N Rua M Raffa RB Diabetes mellitus and Alzheimer's disease: shared pathology and treatment? Brit J Clin Pharmaco 2011 71 365 376 10.1111/j.1365-2125.2010.03830.x PMC3045545 21284695

[B29] Yaffe K Falvey C Hamilton N Schwartz AV Simonsick EM Satterfield S Cauley JA Rosano C Launer LJ Strotmeyer ES Harris TB Diabetes, glucose control, and 9-year cognitive decline among older adults without dementia Arch Neurol 2012 9 1170 1175 22710333 10.1001/archneurol.2012.1117 PMC3752423

[B30] Bomfim TR Forny-Germano L Sathler LB Brito-Moreira J Houzel J-C Decker H Silverman MA Kazi H Melo HM McClean PL Holscher C Arnold SE Talbot K Klein WL Munoz DP Ferreira ST De Felice FG An anti-diabetes agent protects the mouse brain from defective insulin signaling caused by Alzheimer's disease–associated Aβ oligomers J Clin Invest 2012 122 1339 1353 10.1172/JCI57256 22476196 PMC3314445

[B31] McClean P Parthsarathy V Faivre E Hölscher C The diabetes drug Liraglutide prevents degenerative processes in a mouse model of Alzheimer's disease J Neurosci 2011 31 6587 6594 10.1523/JNEUROSCI.0529-11.2011 21525299 PMC6622662

[B32] Moona JH Kima HJ Yanga AH Kima HM Leea BW Kanga ES Leea HC Cha BS The effect of rosiglitazone on LRP1 expression and amyloid β uptake in human brain microvascular endothelial cells: a possible role of a low-dose thiazolidinedione for dementia treatment Int J Neuropsychopharmacol 2012 15 135 142 10.1017/S1461145711001611 22040807

[B33] Younesi E Hofmann-Apitius M A network model of genomic hormone interactions underlying dementia and its translational validation through serendipitous off-target effect J Transl Med 2013 11 177 10.1186/1479-5876-11-177 23885764 PMC3733613

[B34] Simmons LA Dinan MA Robinson TJ Snyderman R Personalized medicine is more than genomic medicine: confusion over terminology impedes progress towards personalized healthcare Pers Med 2012 9 85 91 10.2217/pme.11.86 29783292

[B35] Cascorbi I The promises of personalized medicine Eur J Clin Pharmacol 2010 66 749 754 10.1007/s00228-010-0858-6 20563567

[B36] Bates S Progress towards personalized medicine Drug Discov Today 2010 15 115 10.1016/j.drudis.2009.11.001 19914397

[B37] Nohaile M The biomarker is not the end Drug Discov Today 2011 16 878 883 10.1016/j.drudis.2011.08.011 21888986

[B38] Deyati A Younesi E Hofmann-Apitius M Novac N Challenges and opportunities for oncology biomarker discovery Drug Discov Today 2012 18 614 624 23280501 10.1016/j.drudis.2012.12.011

[B39] Toedter G Li K Sague S Ma K Marano C Macoritto M Park J Deehan R Matthews A Wu GD Lewis JD Arijs I Rutgeerts P Baribaud F Genes associated with intestinal permeability in ulcerative colitis: Changes in expression following infliximab therapy Inflamm Bowel Dis 2012 18 1399 1410 10.1002/ibd.22853 22223479

[B40] Sacristán JA Patient-centered medicine and patient-oriented research: improving health outcomes for individual patients BMC Med Inf Decis Making 2013 13 6 10.1186/1472-6947-13-6 PMC3575265 23294526

[B41] PatientsLikeMe websitehttp://www.patientslikeme.com

[B42] Wicks P Vaughan TE Massagli MP Heywood J Accelerated clinical discovery using self-reported patient data collected online and a patient-matching algorithm Nat Biotechnol 2011 29 411 414 10.1038/nbt.1837 21516084

[B43] Roque FS Jensen PB Schmock H Dalgaard M Andreatta M Hansen T Søeby K Bredkjær S Juul A Werge T Jensen LJ Brunak S Using electronic patient records to discover disease correlations and stratify patient cohorts PLoS Comput Biol 2011 7 e1002141 10.1371/journal.pcbi.1002141 21901084 PMC3161904

[B44] Malhotra A Younesi E Gündel M Müller B Heneka MT Hofmann-Apitius M ADO: a disease ontology representing the domain knowledge specific to Alzheimer's disease Alzheimers Dement 2013 doi: 10.1016/j.jalz.2013.02.009 10.1016/j.jalz.2013.02.009 23830913

[B45] Self-tracking projecthttp://www.quantifiedself.com

[B46] Dunnett SB Björklund A Prospects for new restorative and neuroprotective treatments in Parkinson's disease Nature 1999 399 A32 A39 10392578 10.1038/399a032

[B47] Loscalzo J Kohane I Barabasi AL Human disease classification in the postgenomic era: a complex systems approach to human pathobiology Mol Syst Biol 2007 3 124 17625512 10.1038/msb4100163 PMC1948102

[B48] Committee on A Framework for Developing a New Taxonomy of Disease What would a knowledge network and new taxonomy look like? Towards precision medicine: Building a Knowledge Network for Biomedical Research and a New Taxonomy of Disease 2011 Washington DC: The National Academies Press 35 50 22536618

[B49] Li YY Jones SJM Drug repositioning for personalized medicine Genome Med 2012 4 27 22494857 10.1186/gm326 PMC3446277

[B50] Shaw AT Yasothan U Kirkpatrick P Crizotinib Nat Rev Drug Discov 2011 10 897 898 10.1038/nrd3600 22129984

[B51] Sanseau P Koehler J Computational methods for drug repurposing Briefings Bioinf 2011 12 301 302 10.1093/bib/bbr047 21768130

[B52] Shahid M Shahzad Cheema M Klenner A Younesi E Hofmann-Apitius M SVM based descriptor selection and classification of neurodegenerative disease drugs for pharmacological modeling Mol Inf 2013 32 241 249 10.1002/minf.201200116 27481519

[B53] Butcher EC Berg EL Kunkel EJ Systems biology in drug discovery Nat Biotechnol 2004 22 1253 1259 10.1038/nbt1017 15470465

[B54] Shillingford CA Vose CW Effective decision-making: progressing compounds through clinical development Drug Discov Today 2001 6 941 946 10.1016/S1359-6446(01)01945-6 11546608

[B55] van der Worp HB Howells DW Sena ES Porritt MJ Rewell S O’Collins V Macleod MR Can animal models of disease reliably inform human studies? PLoS Med 2010 7 e1000245 10.1371/journal.pmed.1000245 20361020 PMC2846855

[B56] Seok J Warren HS Cuenca AG Mindrinos MN Baker HV Xu W Richards DR McDonald-Smith GP Gao H Hennessy L Finnerty CC López CM Honari S Moore EE Minei JP Cuschieri J Bankey PE Johnson JL Sperry J Nathens AB Billiar TR West MA Jeschke MG Klein MB Gamelli RL Gibran NS Brownstein BH Miller-Graziano C Calvano SE Mason PH Genomic responses in mouse models poorly mimic human inflammatory diseases Proc Natl Acad Sci U S A 2013 110 3507 3512 10.1073/pnas.1222878110 23401516 PMC3587220

[B57] Geerts H Spiros A Roberts P Carr R Has the time come for predictive computer modeling in CNS drug discovery and development? CPT: Pharmacometrics Syst Pharmacol 2012 1 e16 10.1038/psp.2012.17 23835798 PMC3600733

[B58] Jucker M The benefits and limitations of animal models for translational research in neurodegenerative diseases Nat Med 2010 16 1210 1214 10.1038/nm.2224 21052075

[B59] IMPROVER Systems Biology Verification Challengehttps://www.sbvimprover.com/

[B60] Golubnitschaja O Costigliola V EPMA: General report & recommendations in predictive, preventive and personalised medicine 2012: white paper of the European association of predictive, preventive and personalised medicine EPMA J 2012 3 14 10.1186/1878-5085-3-14 23116135 PMC3485619

